# Cytoskeletal impairment during isoamyl alcohol-induced cell elongation in budding yeast

**DOI:** 10.1038/srep31127

**Published:** 2016-08-10

**Authors:** Wakae Murata, Satoko Kinpara, Nozomi Kitahara, Yoshihiro Yamaguchi, Akira Ogita, Toshio Tanaka, Ken-ichi Fujita

**Affiliations:** 1 Graduate School of Science, Osaka City University, Osaka 558-8585, Japan; 2 Department of Materials Science, National Institute of Technology, Yonago College, Tottori 683-8502, Japan; 3 The OCU Advanced Research Institute for Natural Science and Technology, Osaka City University, Osaka 558-8585, Japan; 4 Research Center for Urban Health and Sports, Osaka City University, Osaka 558-8585, Japan.

## Abstract

Isoamyl alcohol (IAA) induces pseudohyphae including cell elongation in the budding yeast *Saccharomyces cerevisiae*. Detailed regulation of microtubules and actin in developmental transition during cell elongation is poorly understood. Here, we show that although IAA did not affect the intracellular actin level, it reduced the levels of both α- and β-tubulins. In budding yeast, cytoplasmic microtubules are linked to actin via complexes consisting of at least Kar9, Bim1, and Myo2, and reach from the spindle pole body to the cortical attachment site at the bud tip. However, IAA did not affect migration of Myo2 to the bud tip and kept Kar9 in the interior portion of the cell. In addition, bud elongation was observed in Kar9-overexpressing cells in the absence of IAA. These results indicate that impairment of the link between cytoplasmic microtubules and actin is possibly involved in the lowered interaction of Myo2 with Kar9. Our study might explain the reason for delayed cell cycle during IAA-induced cell elongation.

Hypha formation is one of the crucial morphogenetic events not only in most of filamentous fungi but also in yeasts. The budding yeast *Saccharomyces cerevisiae* displays yeast-like growth in the vegetative stage and pseudohyphae formation during nitrogen starvation of diploid cells^1^. It is thought that pseudohyphae formation is a strategy by which a normally sessile organism can forage for microenvironments that are more favourable^2^. Pseudohyphae undergo DNA replication and nuclear division, reminiscent of an aberrant form of the cell division cycle^3^. In addition to typical signalling pathways such as a cAMP signalling pathway and/or a mitogen-activated protein kinase (MAPK) pathway^4^, pseudohyphae formation is also stimulated by G1 cyclin-dependent kinases^5^ and a pheromone-responsive MAPK pathway^1^. The involvement of a transcription factor, Rim101^6^ and a protein required for derepression of glucose-repressed genes, Snf1^7^,^8^ has been reported in pseudohyphae formation. These are supported by the involvement of hundreds of proteins based on genome-wide analysis^9^. On the other hand, a temperature-sensitive mutation of a cyclin B homolog, *SCB1Δ152,* induces the formation of elongated cells^10^.

Heterodimers of α- and β-tubulins are readily polymerized to microtubules and depolymerized. Microtubule-inhibiting drugs restrict polymerisation of tubulin heterodimers^11^,^12^ or limit microtubule depolymerisation^13^, thereby restricting chromosome segregation, and consequently, filamentous growth. Thus, correct microtubule formation can be hypothesised to function in normal cell morphogenesis. Budding yeast cells at different stages in the cell cycle contain three visible F-actin structures: cortical actin patches, polarized actin cables, and a cytokinetic actin ring. While patches and cables appear throughout the cell cycle, the ring is visible for a short period immediately before and during cytokinesis^14^. Bim1 is a microtubule-binding protein^15^ that forms a complex with Kar9^16^ to translocate Kar9 to the cytoplasmic microtubule plus-ends where it binds to Myo2, a class V myosin, resulting in polarized transport of the spindle pole body (SPB) along actin cables to the bud neck^17^. Bud6 is an actin-binding protein and sequentially cues cytoplasmic microtubule capture events at the bud tip followed by capture events at the bud neck, necessary for correct spindle morphogenesis and polarity^18^. Overexpression of the transcriptional activator gene *PHD1* induces pseudohyphae. In the pseudohyphae, the actin cytoskeleton remains polarized throughout bud growth, and short complete and elongated spindles, indicating cell cycle arrest at S, G2, and metaphase, and anaphase, respectively, have been observed^19^.

Fusel alcohols including isoamyl alcohol (IAA) induce filamentous growth under enriched conditions in both haploid and diploid cells^20^. These alcohols are produced by the catabolism of branched-chain amino acids as by-products of alcoholic fermentation^21^. In diploid cells treated with IAA, bud formation is uncoupled from nuclear division^20^. Most reports on IAA-induced pseudohyphae focus on signalling cascades and the cellular response at the initiation or early stage of pseudohyphae formation^1^,^4^.

During this response, yeast cells are thought to exhibit aberrant dynamics of the cytoskeleton, including actin and microtubules. However, the interaction between actin and cytoplasmic microtubules via their binding proteins during IAA-induced cell elongation is currently unclear.

Here, we investigated the molecular function and localization of cytoskeleton-related proteins in IAA-induced elongated cells of haploid *S. cerevisiae* strains. Cell elongation events were elucidated focusing on gene expression and dynamic behaviour of cytoskeletal and associated proteins. Time-lapse imaging was used to reveal cytoskeleton dynamics.

## Results

### IAA induces cell elongation in *S. cerevisiae*

**Figure 1:**
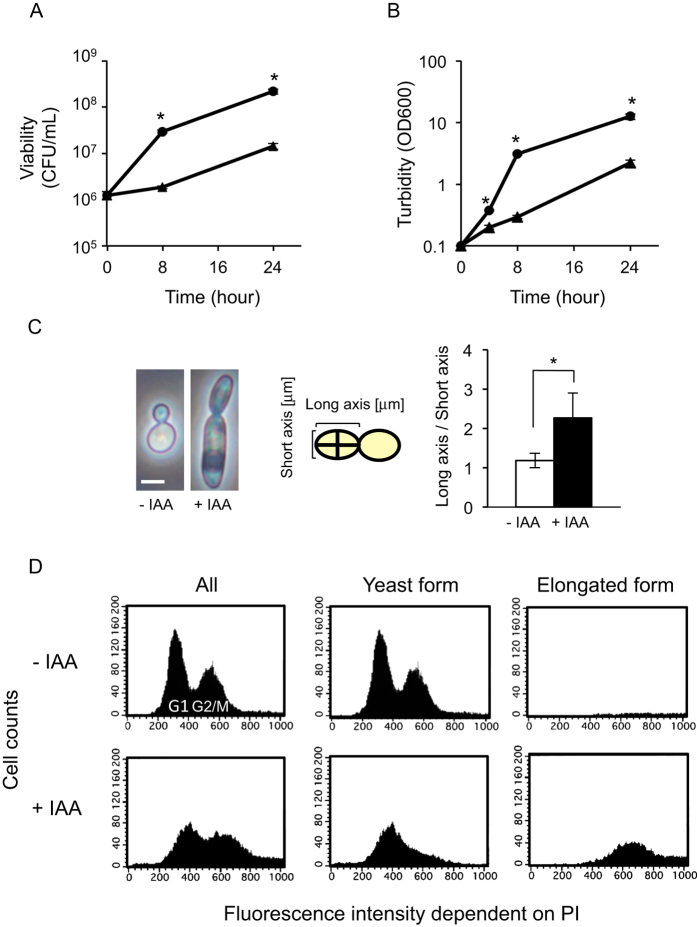
Effects of IAA on viability, proliferation, morphology, and cell cycle. BY4741 cells were incubated in YPD broth with (triangles) or without (circles) 0.5% IAA. (**A**) Viability was evaluated as colony-forming units/mL. Data are means ± standard deviations of triplicate experiments. *P < 0.05 vs. -IAA. (**B**) Cell proliferation was evaluated as turbidity of the cell suspension at 600 nm. Data are means ± standard deviations of triplicate experiments. *P < 0.05 vs. -IAA. (**C**) After 24-h incubation, the length of the long and short axes of cells was measured. Data are means ± standard deviations of triplicate experiments. In each experiment, the cells were selected at random (n > 200). *P < 0.05 vs. -IAA. Bars, 4 μm. (**D**) After 24-h incubation, the propidium iodide-stained cells stained were subjected to cell cycle analysis using FACS. Cells showing sizes similar to those of untreated cells were regarded the yeast form. Cells larger than untreated cells were regarded the elongated form.

The effects of IAA on cellular growth and morphology were analysed by counting colony-forming units (CFU) and measuring culture turbidity, and by microscopic observation, respectively. IAA significantly repressed an increase in viable cell number and culture turbidity during 24-h culture of *S. cerevisiae* BY4741 observed under normal conditions without IAA (Fig. 1A,B). These results indicated that IAA restricted cell division and delayed cell cycle. IAA induced elongation of haploid daughter cells, leading to an increased long-to-short axis ratio (1.9-fold elongation when compared to IAA, Fig. 1C). Similar results were obtained for strains W303-1A and BY23323 (data not shown).

Next, we analysed the effect of IAA on the cell cycle using flow-cytometric analysis of propidium iodide-stained cells, which confirmed that IAA-treated cells contained larger yeast cells than the untreated population (data not shown) and revealed that the elongated cells was induced upon IAA treatment (Fig. 1D). In IAA-treated cells, we observed a decrease in G1-phase and an increase in G2/M-phase cells. In the elongated cells, the cell cycle tended to be arrested at G2/M phase (Fig. 1D), while in the IAA-untreated cells, a decrease in the ratio of cells at G2/M phase was observed. These results were in accordance with IAA-induced restriction of cell division as indicated by the restricted cell growth and increased turbidity as shown in Fig. 1A,B.

### Decrease in α- and β-tubulin levels during cell elongation

**Figure 2:**
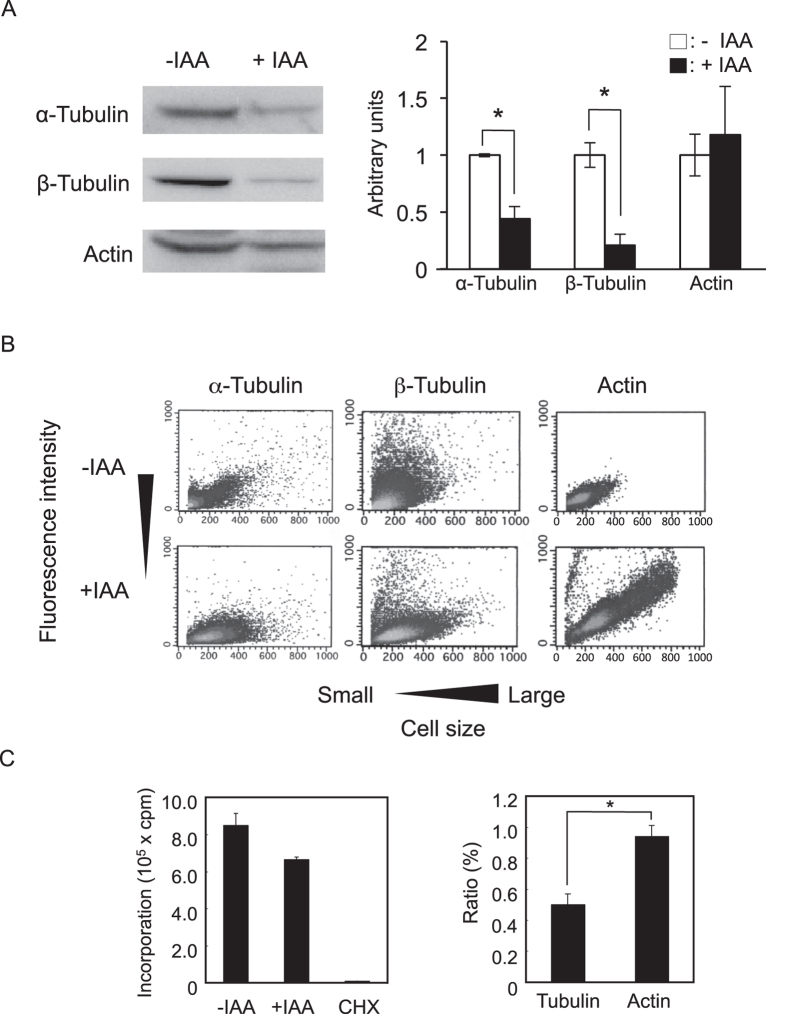
Effects of IAA on intracellular levels and translation of cytoskeleton-related proteins. (**A**) Cell-free extract was obtained from BY4741 cells treated with (+IAA) or without (-IAA) 0.5% IAA for 4 h. (Left panel) Intracellular levels of α-tubulin, β-tubulin, and actin were estimated using western blot analysis. Each lane contained an equal amount of total proteins based on Bradford protein assay. (Right panel) The levels of α-tubulin, β-tubulin, and actin were also quantified using Fujifilm Multi Gauge Version 2.1. Data are means ± standard deviations of triplicate experiments. *P < 0.05 vs. -IAA. (**B**) BY4741 cells incubated with (+IAA) or without (-IAA) 0.5% IAA for 24 h were stained with monoclonal anti-α-tubulin DM1A for α-tubulin, mouse monoclonal, anti-β-tubulin TU27B for β-tubulin, and rhodamine-phalloidin for actin. The cellular content of α-tubulin, β-tubulin, and actin was quantified using FACS. Light grey area indicates high cell density. (**C**) (Left panel) Effect of IAA on translation of total proteins. BY4741 cells were incubated SD medium with (+IAA) or without (-IAA) 0.5% IAA, or with 50 μg/ml cycloheximide (CHX) for 24 h. Incorporation of l-[^35^S]methionine into acid-insoluble fractions was measured after 1 h. Data are means ± standard deviations of triplicate experiments. *P* = 0.058. (Right panel) Effect of IAA on translation of α-tubulin and actin. BY4741 cells were incubated in SD medium with or without 0.5% IAA for 24 h. Incorporation of l-[^35^S]methionine into α-tubulin or actin was evaluated after 1 h. The cell-free extract obtained from the cells was immunoprecipitated with anti-α-tubulin or anti-actin. The ratio of IAA-treated to untreated cells showing l-[^35^S]methionine incorporation is indicated. Data are means ± standard deviations of triplicate experiments. *P < 0.05 vs. actin.

Intracellular levels of total α- and β-tubulins were compared in cells treated with or without IAA using western blot analysis. In IAA-treated cells, both α- and β-tubulins were significantly decreased as compared to control cells (Fig. 2A). However, IAA did not affect the actin level. FACS analysis demonstrated an increase in actin- but not α- and β-tubulin-derived fluorescence intensity with increasing cell size (Fig. 2B), confirming that IAA treatment reduced the relatively cellular amount of α- and β-tubulins. *S. cerevisiae* has four tubulin genes; two genes for α-tubulin (*TUB1* and *TUB3*), one for β-tubulin (*TUB2*), and one for γ-tubulin (*TUB4*). Deletion of *TUB1* is lethal because it provides approximately 90% of α-tubulin of the cells^22^. Although *TUB3* is minor, it can support viability in the absence of *TUB1* if *TUB3* is expressed at a sufficiently high level^23^. Thus, it has been concluded that these two proteins function interchangeably^23^. γ-Tubulin is involved in nucleating microtubules from both the cytoplasmic and the nuclear faces of the SPB. However, γ-tubulins are not a component of the microtubule fibre; microtubule nucleation from γ-tubulin complexes is essential for microtubule formation^24^. *TDH1* encoding glyceraldehyde-3-phosphate dehydrogenase was used as a positive control of RT-PCR analysis. Additionally, we checked the effect of IAA on transcription of *ACT1* encoding another cytoskeleton protein, actin. In RT-PCR analysis, a slight decrease in the expression of *TUB1* was observed in cells treated with IAA (Supplementary Fig. S1). However, the expression levels of *TUB2, TUB3, TUB4* and *ACT1* genes were almost identical in treated and untreated yeast cells (Supplementary Fig. S1). These results indicated that α- and β-tubulin levels are independently regulated and aberrant cytoskeletal protein levels possibly affect yeast morphology.

To gain further insight into the mechanism underlying tubulin loss, we evaluated the effect of IAA on translation of total proteins, α-tubulin, and actin. Total protein translation was estimated by measuring the incorporation of l-[^35^S]methionine into acid-insoluble fractions. A typical inhibitor of protein biosynthesis, cycloheximide, was used as a positive control. This antibiotic completely inhibited nonspecific protein translation (Fig. 2C, left panel) and induced 48% growth inhibition at 0.1 μg/mL, while no morphological changes were observed in the treated cells (data not shown). The incorporation seemed to be slightly, but not significantly (*P* = 0.058), restricted by IAA treatment (Fig. 2C, left panel). Immunoprecipitation assay indicated that the uptake of l-[^35^S]methionine into α-tubulin was significantly more inhibited than that into actin (Fig. 2C, right panel), for which the effect of IAA on total protein translation was not significant (Fig. 2C, left panel). The decrease in α-tubulin might partly explain the IAA-induced decrease in α-tubulin-specific translation. Thus, IAA is probably a weak inhibitor of total protein biosynthesis, particularly for α-tubulin.

Furthermore, we determined the relative transcription levels of genes involved in mRNA modification and translation, and of the *UBI4* gene encoding ubiquitin, which are normally constitutively expressed, by using RT-PCR. A translation initiation factor eIF4E^25^,^26^ has been reported to be required for pseudohyphae formation under nitrogen-limiting conditions. eIF4E recognizes the mRNA cap structure and also interacts with the ‘multi-adaptor’ proteins eIF4G^27^. Therefore, we examined the transcription levels of the genes encoding eIF4E, poly(A)-binding protein (PABP), Eap1, and Caf20, which are related to mRNA modification^28^, of the genes encoding eIF2B and eIF5A (Supplementary Fig. S1), which are involved in translation^29^,^30^, and of *UBI4* encoding ubiquitin^31^, which all are normally constitutively expressed. RT-PCR analysis revealed a decrease in the expression of these genes in cells treated with IAA, but a slight decrease in expression of eIF2B and a slight increase in expression of *UBI4* (Supplementary Fig. S1). These results suggested that IAA treatment might result in decreased levels of total proteins.

### Localization of tubulins and actin during cell elongation

**Figure 3:**
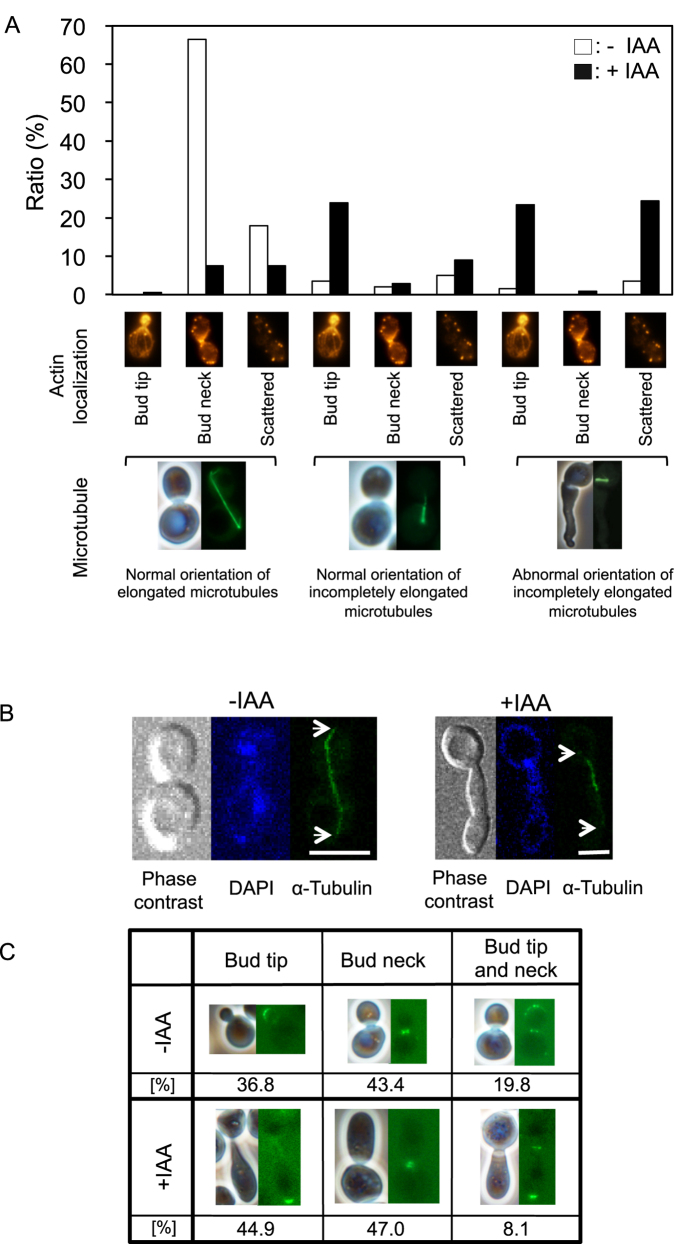
Localization of microtubules and actin during cell elongation. (**A**) BY23323 cells were incubated with (+IAA) or without (-IAA) 0.25% IAA. Microtubules and actin were visualized based on fluorescence derived from Tub1-GFP and rhodamine-phalloidin staining, respectively. More than 200 cells were randomly selected from daughter cells that had grown sufficiently big in the control treatment or from elongated cells in IAA treatment. The selected cells were observed and classified. Actin: left, actin patches were observed at the bud tip; centre, actin ring was formed at the bud neck; right, actin patches were scattered throughout the cytoplasm. Microtubules: left, normal orientation of elongated microtubules; centre, normal orientation of incompletely elongated microtubules; right, abnormal orientation of incompletely elongated microtubules. (**B**) BY23323 cells incubated with (+IAA) or without (-IAA) 0.25% IAA for 8 h were scanned in 11 steps with 0.5-μm step size. DNA was stained with DAPI. Arrows indicate the plus ends of microtubules. Bars, 4 μm. (**C**) EY0986/GFP-Bud6 cells were incubated with (+IAA) or without (-IAA) 0.5% IAA for 8 h. Bud6 was visualized based on fluorescence derived from GFP-Bud6. More than 200 cells randomly selected from budded cells in control treatment or from elongated cells in treatment with IAA were observed and classified.

We visualized the localization of microtubules and actin in IAA-induced elongated cells by measuring the fluorescence of Tub1-GFP and the staining intensity of the actin-binding rhodamine-phalloidin, respectively. For this assay, BY23323 cells, which are more sensitive to IAA than other strains (data not shown), were treated with 0.25% IAA. Cytoplasmic microtubules were elongated and actin was localized at the bud neck in 66.5% of control cells (Fig. 3A). Nucleospindles showed abnormal orientation in 48.8% of elongated cells and actin was localized at the bud tip in 47.9% of these cells. Even if microtubules were properly elongated in IAA-treated cells (15.5% of total cells), a decrease in the rate of cells in which actin localized at the bud neck was observed. This result might explain the fact that cells entered cytokinesis when daughter cells grew sufficiently big.

In fluorescence microscopy, full-length cytoplasmic microtubules were not always observed because microtubules at different depths do not come into focus due to their thickness. We confirmed the localization of full-length microtubules from spindle pole body (SPB) to bud tip on different levels using a confocal microscope. In IAA-induced elongated cells, microtubules were not elongated from the SPB to the cell end or cortical attachment site at the bud tip (Fig. 3B).

### Localization and transcription levels of actin- and tubulin-binding proteins during cell elongation

Bud6 is observed only during bud formation and cytokinesis^18^, while actin is observed throughout cell cycle^14^. Figure 3C shows the colocalization of actin and GFP-Bud6 during bud formation and cytokinesis (Fig. 3A,C). IAA elevated the ratio of cells with GFP-Bud6 expression at the bud tip, while reducing the ratio of cells in which GFP-Bud6 localized to the bud tip and neck, indicating IAA-induced delay of cytokinesis initiation.

**Figure 4:**
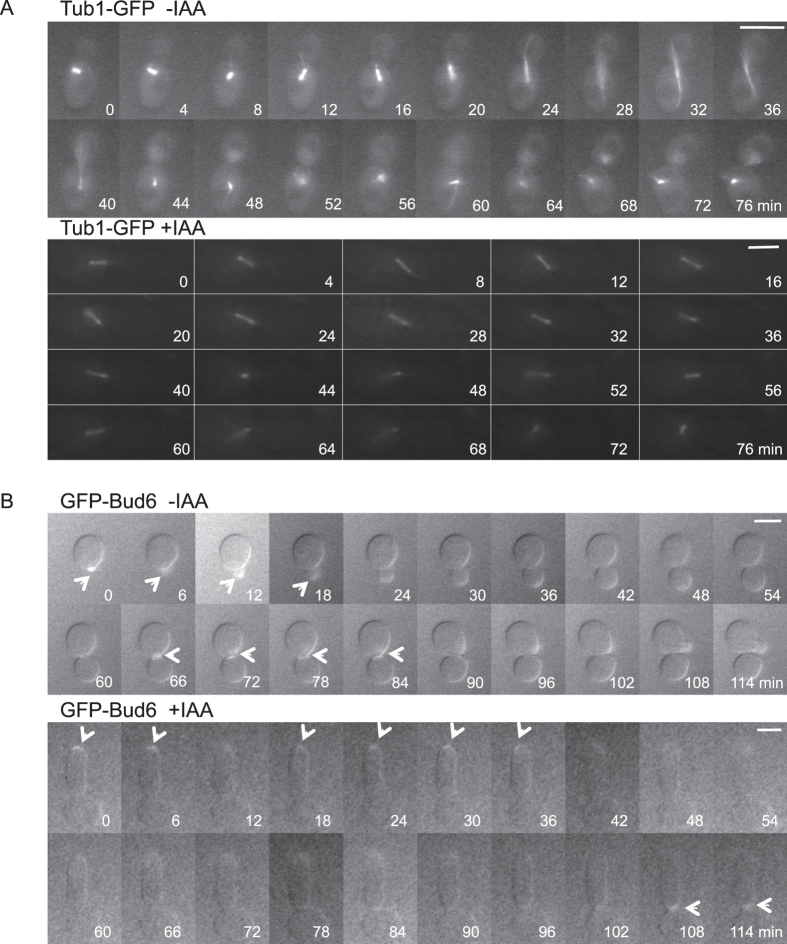
Time-lapse observation of microtubules and Bud6. Among images obtained every 2 min, and representative images at 4-min intervals for Tub1-GFP and at 6-min intervals for GFP-Bud6 are shown. Arrows indicate fluorescent signal. Bars, 4 μm. (**A**) BY23323 cells were incubated with (+IAA) or without (-IAA) 1% IAA on agar plates. Microtubules were visualized based on fluorescence derived from Tub1-GFP. (**B**) EY0986/GFP-Bud6 cells were incubated with (+IAA) or without (-IAA) 1% IAA on agar plates. Bud6 was visualized based on fluorescence derived from GFP-Bud6.

Time-lapse imaging of control and IAA-treated cells expressing Tub1-GFP and GFP-Bud6 confirmed that Bud6 migrates similarly to actin. Figure 4 and Supplementary Movies 1,2,3,4 show the dynamics of Tub1-GFP and GFP-Bud6 in representative single living cells. In normally budding cells, the nucleospindle, visualised by Tub1-GFP, located to the bud neck at the start of bud formation. Until cytoplasmic microtubules captured the bud tip, the orientation of the nucleospindle was not fixed, but rather seemed to be oscillating. Immediately after cytoplasmic microtubules were elongated up to the bud tip, the nucleospindle was properly oriented; it elongated alongside the long axis of the cells (Fig. 4A). However, in IAA-induced elongated cells, nucleospindles were observed but cytoplasmic microtubules were not, indicating inadequate elongation. In addition, oscillation of the nucleospindle continued for a longer time. Moreover, the migration of GFP-Bud6 in elongated cells was slower than that in normally budding cells, and the transition of Bud6 from bud tip to bud neck was observed after 108 min in elongated cells (Fig. 4B).

**Figure 5:**
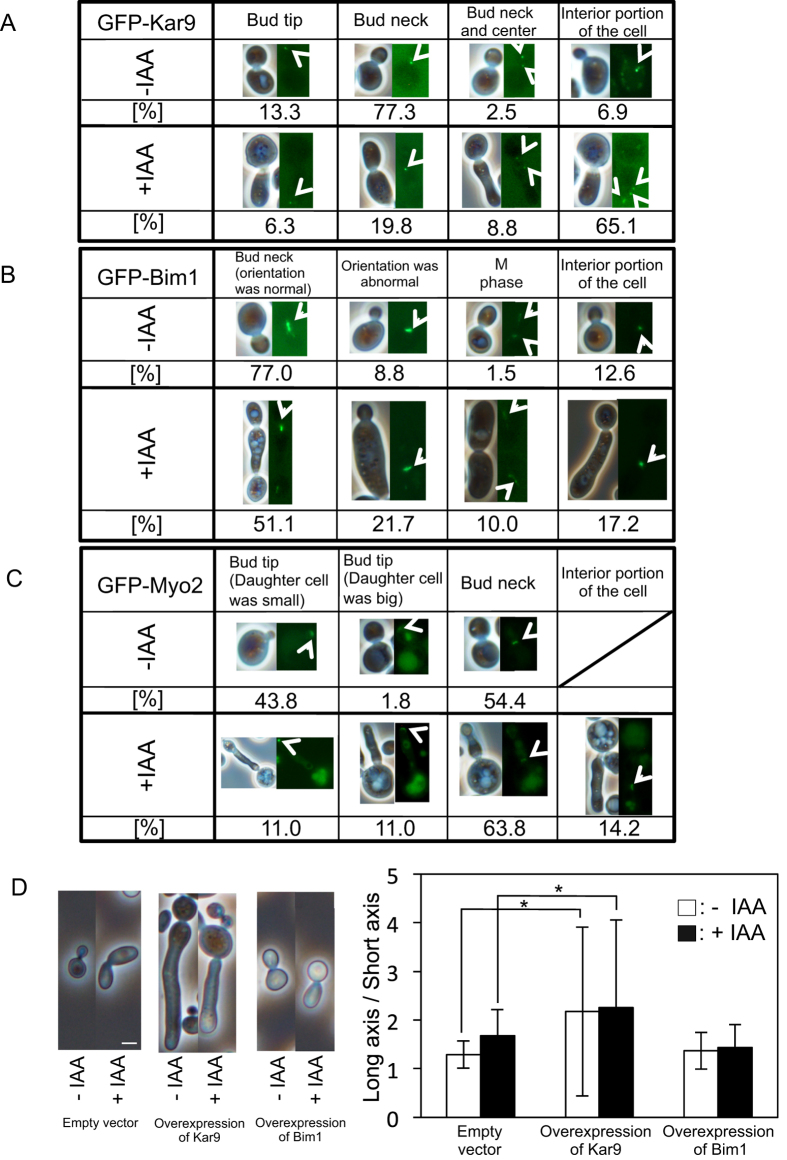
Localization of Kar9, Bim1 and Myo2, and cell morphology of Kar9- and Bim1*-*overexpressing cells. (**A–C**) EY0986/GFP-Kar9, EY0986/GFP-Bim1, or BY24051 cells for visualization of Kar9, Bim1, or Myo2, respectively, were incubated with (+IAA) or without (-IAA) 0.5% IAA for 8 h. The proteins were visualized based on fluorescence derived from GFP-fusion proteins. More than 200 cells were selected at random, from budded cells in treatment without IAA or from elongated cells in treatment with IAA. The cells were observed and then classified. Arrows indicate localization sites of GFP-fusion proteins. (**D**) pYES2 empty vector-harbouring and Kar9*-* and Bim1*-*overexpressing cells were incubated in SG medium with (+IAA) or without (-IAA) 0.25% IAA for 24 h. (Left panel) Morphologies of representative cells are shown. Bar, 4 μm. (Right panel) The lengths of long and short axes of randomly selected cells were measured. Data are means ± standard deviations of triplicate experiments. In each experiment, the budding cells were randomly selected (n > 200). *P < 0.05 vs. empty vector.

After 8-h incubation with or without IAA of strains expressing GFP-Kar9, GFP-Bim1, and GFP-Myo2, the localization of these proteins was assessed (Fig. 5A–C). In 77.3% of normally budding cells, Kar9 was localized at the bud neck (Fig. 5A). In contrast, Kar9 was localized at the bud neck in only 19.8% of IAA-treated cells, while in 65.1%, it localized to the interior of the cells. In 77% of normally budding cells, Bim1 localized to the nucleospindle, which was oriented normally at the bud neck (Fig. 5B). An increase in cells with abnormal localization of Bim1 was observed when daughter cells sufficiently elongated upon treatment with IAA (Fig. 5B) indicating abnormal orientation of spindle. The ratio of cells in which Myo2 localized to the bud tip in elongating daughter cells increased upon treatment with IAA (Fig. 5C).

**Figure 6:**
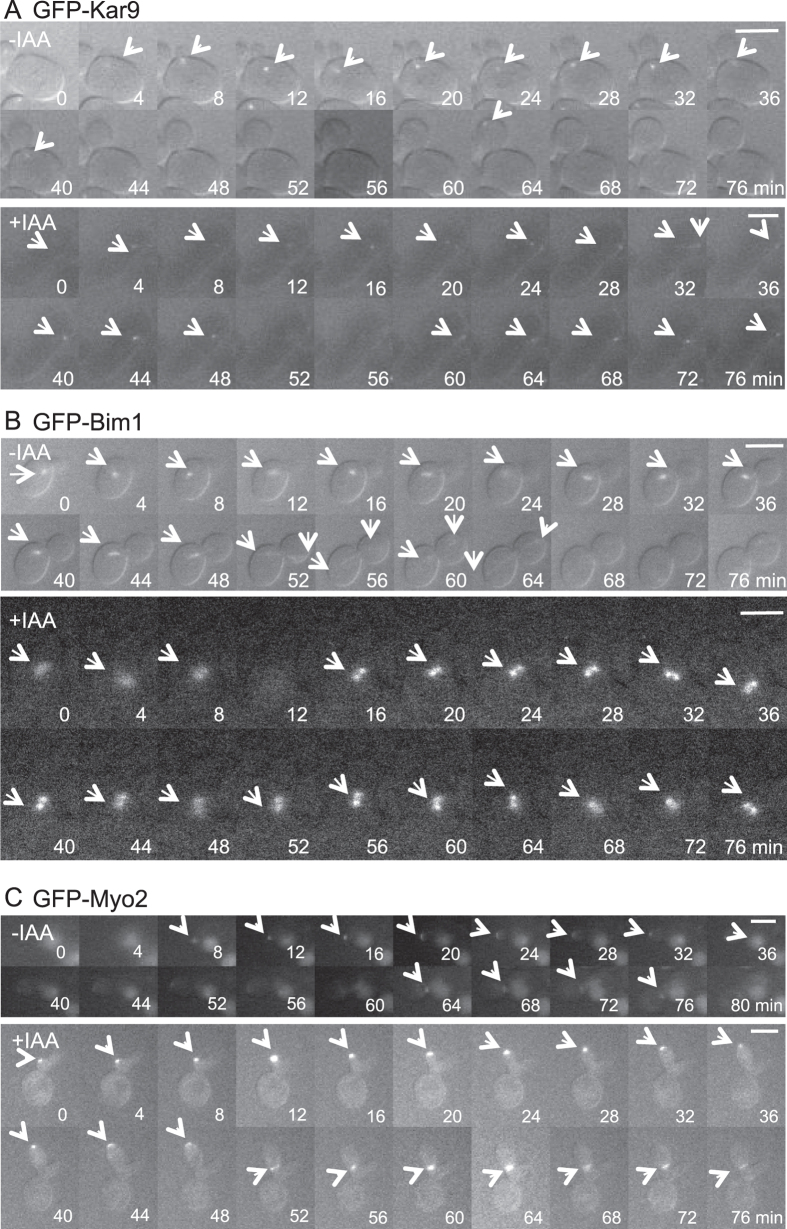
Time-lapse observation of Kar9, Bim1, and Myo2. During time-lapse observation, EY0986/GFP-Kar9 (**A**) EY0986/GFP-Bim1 (**B**) and BY24051 (**C**) cells were incubated with (+IAA) or without (-IAA) 1% IAA. The proteins were visualized based on fluorescence derived from GFP-fusion proteins. Images were obtained every 2 min, and representative images at 4-min intervals are shown (see also Supplementary Movie 5,6,7,8,9,10). Arrows indicate localization sites of GFP-fusion proteins. Bars, 4 μm.

Figure 6 and Supplementary Movies 5,6,7,8,9,10 show the dynamic behaviour of GFP-Kar9, GFP-Bim1, and GFP-Myo2 during bud formation using time-lapse observation. In untreated cells, Kar9 was localized at the bud neck during early stages of bud formation. After the bud was fully formed, Kar9 localized to the bud tip and then disappeared (Fig. 6A). These results were in good agreement with previously reported results^32^. In contrast, in IAA-induced elongated cells, Kar9 did not migrate from the bud neck to the tip but remained in the interior of the bud. Bim1 resembled Kar9 in dynamic behaviour regardless of IAA treatment, indicating that IAA does not affect complex formation of Kar9 and Bim1 (Fig. 6A,B). Both proteins localized to the bud neck in approximately 40 min in normally budding cells and subsequently migrated to the bud tip, after which cell division was completed. However, in elongated cells, the complex of Kar9 and Bim1 did not readily migrate to the bud tips. Bim1 also resembled Tub1 in behaviour. Myo2 migrated to the bud tip even in elongated cells (Fig. 6C).

Next, we examined the effect of overexpression of Kar9 and Bim1 on cell morphology. Cell turbidity was significantly elevated after 24-h culture without IAA in Kar9- and Bim1-overexpressing strains, while 0.5% IAA restricted the growth of both strains (Supplementary Fig. S2). Cells overexpressing Kar9 were elongated as compared to the parental strain regardless of IAA (Fig. 5D). However, cells overexpressing Bim1 were not elongated. On the other hand, cell morphology of Kar9-deficient strains resembled that of the parental strain (Supplementary Fig. S3).

## Discussion

We found that IAA induced cell elongation and caused cell cycle arrest or delay at G2/M and abnormal nuclear division in *S. cerevisiae* (Fig. 1C,D), in accordance with previously reported results^3^. Additionally, intracellular levels of α- and β-tubulins were lower in the elongated cells (Fig. 2A,B). In the fungus *Aspergillus nidulans*, the amino acid analogue l-2,5-dihydrophenylalanine caused growth inhibition with loss of microtubules due to decreased α- and β-tubulins^33^. 1-Methyl-4-phenyl-1,2,3,6-tetrahydropyridine and rotenone strongly depolymerized microtubules and accelerated tubulin degradation in the rat brain^34^. Microtubules, especially spindle microtubules, are widely recognized to be important in cell cycle progression from metaphase to anaphase^35^. The spindle position checkpoint blocks mitotic exit and cytokinesis in the case of mitotic spindle misalignment^36^. We thought that IAA could not directly interact with microtubules. Because of many signaling pathways are involved in pseudohyphae formation including cell elongation^9^, IAA might act on the upstream cascade of the pathway thereby indirectly affecting microtubule dynamics. A decrease in tubulin levels was also observed in IAA-treatment. On the other hand, we observed cell morphology of a strain with a decrease in gene expression levels of *TUB2*, which showed decreased abundance by mRNA perturbation (DAmP)^37^. Ratio of long / short axis of cells was similar in the *TUB2*-DAmP and its parent strains, or cell elongation was rather suppressed by IAA treatment (data not shown). These results suggested that a decrease in tubulins and/or microtubules was not directly involved in a delay in the cell cycle. Involvement of many genes and hundreds of proteins in IAA-induced pseudohyphae has been reported^1^,^9^. Therefore, we could not conclude that the phenomenon was attributed to a certain specific signalling pathway based on the results obtained with this study.

On the other hand, our study revealed that the intracellular level of actin, which generally guides the orientation of microtubule growth, was not drastically changed by IAA. While in budding yeast cells, cortical actin patches and polarized actin cables are visible throughout cell cycle, the cytokinetic actin ring is visible for a short period before and during cytokinesis^13^. In IAA-induced elongated cells, nucleospindle orientation was aberrant and cytoplasmic microtubules did not fully elongate from the SPB to the cortical attachment site at the bud tip (Figs 3 and 4). These phenomena were rarely observed in normally budding control cells. Moreover, even if IAA-treated daughter cells grew sufficiently large, the actin patches remained around the bud tip, which was associated with cell cycle delay in half of the cells. In the rest of the cells, the actin contractile ring required for cytokinesis was formed normally (Figs 3 and 4). In normally budding cells, actin was first localized at the bud tip and the cytoplasmic microtubules were elongated to the bud tip along actin cables or the actin patches at the bud tip^13^,^38^. Cytokinesis was then finished at telophase. Cyclins regulate cell cycle progression by binding to and activating cyclin-dependent kinases^39^, and mitotic exit regulation involves inactivation of mitotic kinases and activation of counteracting protein phosphatases^40^. In addition, the nucleospindle plays a major role in cell cycle progression^41^,^42^. However, in this study, cytoplasmic microtubules did not fully elongate from the SPB to the end of mother cells and the tip of daughter cells even if the nucleospindle was correctly orientated. In addition, in IAA-treated cells with fully elongated cytoplasmic microtubules, the microtubules were thinner than those in untreated cells (Fig. 3B).

From the above results, we inferred that the plus ends of microtubules elongate to the cortical attachment site at the bud tip, where they recruited a complex consisting of Bim1, Kar9 and Myo2, and that the binding of the plus ends to the cortical attachment site via Cdc42 complex triggers actins to migrate from bud tip to bud neck. Control of cytokinesis has been reported to involve cyclin-dependent kinases^43^. To our knowledge, the detailed cellular process and signalling pathway of contractile ring formation are not yet understood. In support of our hypothesis, cells with insufficiently elongated cytoplasmic microtubules showed decreased contractile ring formation (Fig. 3). Cyclin and the mitotic exit network are very important for cell cycle progression^44^,^45^. We hypothesize that cytoplasmic microtubule elongation reaching the cortical attachment site or subsequent binding of Kar9–Bim1 complex to the site also play an important role in cell cycle progression and formation of the contractile ring. Based on our combined findings, we reasoned that abnormality of cytoplasmic microtubules caused elongated cells. In particular, we assumed that IAA treatment resulted in abnormalities in microtubule-associated proteins, which primarily bind to actins. Therefore, we analysed their localization in elongated cells using mainly florescence microscopy.

Migration of Bim1, which normally binds to the microtubule plus ends, was aberrant. Moreover, Kar9 remained in the cell interior for a long time during cell elongation, showing so-called ‘oscillation’ while searching for its final destination (Supplementary Movie 6). During normal bud formation, these proteins migrate to the bud tip as the bud grows. The direct interaction of Myo2 with Kar9 is possibly a signal to advance cell cycle. However, Myo2 remained at the bud tip after elongation of the daughter cell in the presence of IAA (Figs 5C and 6C). From these results, we infer that inability of Myo2 to associate with Kar9 at least inhibits the connection between cytoplasmic microtubules and actin. Furthermore, overexpression of Kar9 induced cell elongation regardless of treatment with IAA (Fig. 5D). Overexpression of Kar9 might induce scattered localization of Kar9, thereby increasing target sites for Bim1 binding and hindering the interaction between Kar9 and Myo2, resulting in continuation of bud elongation. However, the reason for the lower interaction of Kar9 with Myo2 in elongated cells induced by both IAA and by overexpression of Kar9 remains unclear.

We suggest that IAA-induced elongated cells depends on abnormal orientation of the nucleospindle and the inhibition of cytoplasmic microtubule elongation. Oscillating or abnormal spindle microtubule orientation was also observed in normally budding cells. However, in this case, time-lapse observation revealed that this oscillation was only brief and related to a normal event needed for searching correct cell polarity for cell division. We hypothesize that in elongated cells, the nucleospindle does not seamlessly recognize the correct orientation, leading to cell cycle delay. As a result, the bud continues extensional growth or daughter cells elongate although cell cycle is arrested. We suggest that, as actin remains located at the bud tip in elongated cells, probably due to lower association of Kar9 with Myo2, daughter cells continue to elongate.

In conclusion, we put forth a new hypothesis relating to cytoskeletal dynamics and function during IAA-induced cell elongation in *S. cerevisiae*. Properly elongated cytoplasmic microtubules are at least involved in cell or bud elongation. Namely, continuing cell elongation could be explained by defective interaction of Kar9 with Myo2. These events possibly result in pseudohyphae formation.

## Methods

### Yeast strains

The strains used in this study are listed in Supplementary Tables S1. Unless otherwise stated, yeast cells were grown in YPD broth, consisting of 1% yeast extract, 2% peptone, and 2% glucose. For preparation of agar plates, the medium was supplemented with 2% agar. Yeast cultures were grown at 30 °C unless otherwise indicated.

For overexpression of Kar9 and Bim1, the corresponding genes were cloned into shuttle vector pYES2 (Invitrogen, Carlsbad, CA, USA). The PCR primers used for amplification of the genes are listed in Supplementary Table S2. pYES2 vector was digested with *Sac*I and *Xba*I. The PCR products were incorporated into pYES2 by the SliCE method^46^. Both gene constructs and pYES2 empty vector (negative control) were transformed into BY4741 cells. The transformed cells were maintained on SC-U plates (2% glucose, 0.67% yeast nitrogen base without amino acids, 0.14% dropout mix, 0.008% tryptophan, 0.04% leucine, 0.01% histidine, and 2% agar).

### Culture and treatments

All yeast cells tested were maintained on YPD agar plates. Cells taken directly from the plates were pre-incubated in 5 mL of YPD broth at 30 °C with shaking for 16 h. After pre-incubation, the cells were inoculated into 5 mL of YPD broth (10^6^ cells/mL) and grown with shaking in the presence or absence of IAA. IAA was added at 0.5% (v/v) for BY4741, EY0986/GFP-Kar9, and EY0986/GFP-Bim1, BEY0986/GFP-Bud6, and at 0.25% for W303-1A, BY23323, and BY24051 strains, respectively. Overexpressing strains were maintained on SC-U agar plates. Cells directly taken from the plates were pre-incubated in 1.5 mL of SC-U broth at 30 °C with shaking for 16 h. After pre-incubation, cells (10^6^ cells/mL) were inoculated into 50 mL of SC-U broth and grown with shaking until the logarithmic growth phase was reached. The cells were washed with deionized water, inoculated into 10 mL of SG-U (2% galactose, 0.67% yeast nitrogen base without amino acids, 0.14% dropout mix, 0.008% tryptophan, 0.04% leucine, and 0.01% histidine) broth in the presence or absence of 0.25% IAA, and grown with shaking for 24 h.

### Flow cytometry

BY4741 cells were grown with shaking in the presence or absence of 0.5% IAA at 30 °C for 24 h prior to analysis by flow cytometry using a FACScan (Becton Dickinson, Franklin Lakes, New Jersey, USA). Data were analysed using CellQuest. For analysis of DNA content, the cells were fixed with 95% ice-cold ethanol at 4 °C for 12 h and then incubated with 0.1 mg/mL ribonuclease (Nippon Gene) at 37 °C for 2 h. Propidium iodide (2 μg/mL; Sigma) was added to each cell suspension. For analysis of microtubule content, cells were prepared as described previously^47^. Mouse monoclonal anti-α-tubulin (Sigma, clone: DM1A) diluted at 1:500 and Alexa Fluor 488 goat anti-mouse IgG (H+L) (Molecular Probes) at 1:200 were used as a primary and secondary antibodies, respectively. For analysis of actin content, cells were fixed with 3.7% formaldehyde at room temperature for 30 min and then incubated with 50 mM 2-mercaptoethanol and 1% Triton X-100 at room temperature for 1 h. The cells were stained with 0.165 μM rhodamine-phalloidin at room temperature for 30 min.

### Western blot analysis

Cells grown in the presence or absence of IAA at 30 °C for 4 h were harvested. Whole protein extracts were prepared and protein concentrations were quantified by the Bradford assay^48^. Western blot analysis was performed as described^34^. Primary antibodies used were mouse monoclonal anti-α-tubulin (Sigma) diluted at 1:500, mouse monoclonal anti-β-tubulin TU27B kindly provided by B. R. Oakley (The Ohio State University, Columbus, OH) diluted at 1:200, and rabbit monoclonal anti-actin (Sigma) diluted at 1:100. Secondary antibodies used were goat anti-mouse IgG (H+L), HRP conjugate (Promega) diluted at 1:1,000, horseradish peroxidase-conjugated goat anti-mouse IgG (DAKO, Glostrup, Denmark) diluted at 1:1,500 and horseradish peroxidase-conjugated goat anti-rabbit IgG (H+L) horseradish peroxidase conjugate (Bio-Rad) diluted at 1:3,000 for anti-α-tubulin, anti-β-tubulin, and anti-actin, respectively.

### Incorporation of radioactive methionine into total proteins, α-tubulin, and actin

For incorporation of l-[^35^S]-methionine into total proteins, BY4741 cells (10^7^ cells/mL) were incubated in SD medium lacking methionine supplemented with 0.5% IAA or 50 μg/mL cycloheximide (Wako). Radiolabeling was carried out as described previously^49^. Cells were withdrawn at 0 and 1 h after addition of 2 μCi/mL of l-[^35^S]-methionine (Institute of Isotopes) to the culture. Incorporation of l-[^35^S]-methionine was then stopped by adding 5% trichloroacetic acid. Incorporation of l-[^35^S]-methionine into α-tubulin and actin was analysed using immunoprecipitation. Immunoprecipitation was done using the cells radiolabelled with 2 μCi/mL of l-[^35^S]-methionine for 1 h as the following method. Cells were suspended into an extraction buffer (0.5 M NaCl, 50 mM Tris-HCl (pH 7.5), 10 mM EDTA, 2 mM EGTA, 1% Triton X-100, 1 mM PMSF, 10 μg/mL leupeptin, 10 μg/mL pepstatin, and 10 μg/mL aprotinin), and homogenised by vortexing with glass beads. The supernatants were incubated with mouse monoclonal anti-α-tubulin (Sigma, clone: DM1A) or rabbit monoclonal anti-actin (Sigma) at 4 °C for 17 h, and then incubated with protein G on Sepharose 4B fast flow (equilibrated in the same buffer) (Sigma) at 4 °C for 2 h. The gel was dried with RapiDry mini AE-3711 (ATTO) and exposed to a Fuji imaging plate (BAS-IP TR2040, Fujifilm). The imaging plate was scanned using Science Imaging System FLA-3000 (Fujifilm).

### Fluorescence microscopy

GFP-fusion proteins were used for protein visualization. Actin was stained using rhodamine-phalloidin. Yeast cells were stained using the same method as that used for flow cytometry as described above. The cells were observed with a standard fluorescence microscope (BX53; Olympus, Tokyo, Japan) equipped with a digital camera system DP73 (Olympus) and with a confocal laser-scanning microscope (DM6000B; Leica, Wetzlar, Germany) with digital camera system TCS SP8 (Leica). For confocal imaging, each cell was scanned in 11 steps with 0.5-μm step size, covering the entire region of interest from top to bottom in the yeast cells. For visualization of GFP-fusion proteins, excitation wavelength at 460 nm and emission wavelength at 515 nm were used. For actin, excitation at 540 nm and emission at 565 nm were used. For α-tubulin, excitation at 495 nm and emission at 545 nm were used. For DAPI, excitation at 415 nm and emission at 500 nm were used.

### Time-lapse imaging

Time-lapse observation was performed as reported^50^. Cells treated with or without 0.5% IAA for EY0986/GFP-Kar9, EY0986/GFP-Bim1, BEY0986/GFP-Bud6, and 0.25% IAA for BY23323 and BY24051 strains in 5 mL of YPD broth with shaking at 30 °C for 16 h prior to time-lapse observation in YPD agar with or without 1% IAA. Cells (1.0 × 10^7^) were spread onto YPD agar plates. Images were obtained every 2 min.

### RT-PCR analysis

Gene expression was relatively quantified using RT-PCR in BY4741 cells treated with or without IAA in YPD broth with shaking at 30 °C for 4 h. Total mRNA was isolated from the cells using the RNeasy Mini Kit (QIAGEN) and 0.5–5 μg was used for cDNA synthesis using ReverTra Ace (TOYOBO). PCR was carried out using a standard quantitative amplification protocol. The primers used in this study are listed in Supplementary Table S2.

### Statistical analysis

Group means were compared using Student’s *t*-test, in which a *P*-value < 0.05 was considered statistically significant.

## Additional Information

**How to cite this article**: Murata, W. *et al*. Cytoskeletal impairment during isoamyl alcohol-induced cell elongation in budding yeast. *Sci. Rep.* **6**, 31127; doi: 10.1038/srep31127 (2016).

## Supplementary Material

Supplementary Information

Supplementary Movie 1

Supplementary Movie 2

Supplementary Movie 3

Supplementary Movie 4

Supplementary Movie 5

Supplementary Movie 6

Supplementary Movie 7

Supplementary Movie 8

Supplementary Movie 9

Supplementary Movie 10
