# Supplementary material for: Cytoskeletal impairment during isoamyl alcohol-induced cell elongation in budding yeast
Source: Sci Rep. 2016 Aug 10;6:31127. doi: 10.1038/srep31127 (PMC4979020; doi:10.1038/srep31127)
Supplement: Supplementary Information [file srep31127-s1.pdf]

SUPPLEMENTARY INFORMATION

Cytoskeletal impairment during isoamyl alcohol-induced cell elongation in budding yeast

Wakae Murata[1,2], Satoko Kinpara[1], Nozomi Kitahara[1], Yoshihiro Yamaguchi,[1,3] Akira Ogita[1,4], Toshio Tanaka[1], Ken-ichi Fujita[1]*

[1]Graduate School of Science, Osaka City University, Osaka 558-8585, Japan.

[2]Department of Materials Science, National Institute of Technology, Yonago College, Tottori 683-8502, Japan.

[3] The OCU Advanced Research Institute for Natural Science and Technology, Osaka City University, Osaka 558-8585, Japan.

[4]Research Center for Urban Health and Sports, Osaka City University, Osaka 558-8585, Japan.

*E-mail: kfujita@sci.osaka-cu.ac.jp

Supplementary Video S1 and S2. Effect of IAA on microtubules under time-lapse observation. BY23323 cells were incubated with (Video S1) or without (Video S2) 1% IAA on YPD agar plates. Microtubules were visualized based on fluorescence derived from Tub1-GFP. Time-lapse images were obtained every 2 min.

Supplementary Video S3-S10. Effect of IAA on Bud6, Kar9, Bim1, and Myo2 under time-lapse observation. EY0986/GFP-Bud6 cells were incubated with (Video S3) or without (Video S4) 1% IAA, EY0986/GFP-Kar9 cells were incubated with (Video S5) or without (Video S6) 1% IAA, EY0986/GFP-Bim1 cells were incubated with (Video S7) or without (Video S8) 1% IAA, and BY24051 cells for Myo2 were incubated with (Video S9) or without (Video S10) 1% IAA on YPD agar plates. The proteins were visualized based on fluorescence derived from GFP-fusion proteins. Time-lapse images were obtained every 2 min.

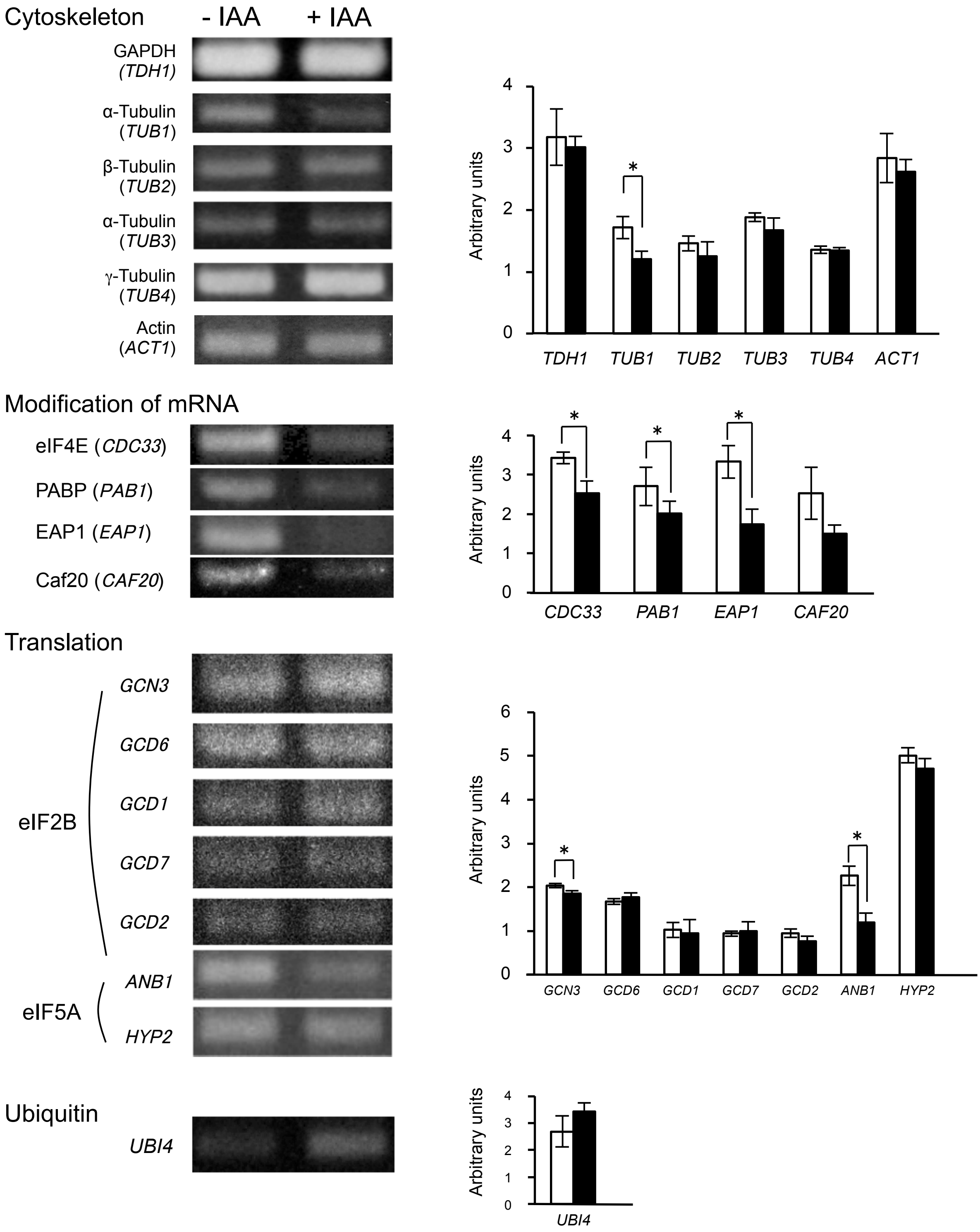

Supplementary Figure S1. Effect of IAA on the expression of genes related to cytoskeleton, translation, modification of mRNA and of ubiquitin.
BY4741 cells were incubated with (+IAA) or without (-IAA) 0.5% IAA for 4 h. (left) Fragments of indicated genes were amplified by RT-PCR, electrophoresed on a 2% agarose gel, and stained with GelRed. *TDH1* for glyceraldehyde-3-phosphate dehydrogenase was used as a positive control. (right) The results quantitated are shown. Open, -IAA; Closed,+IAA. *$P < 0.05$ vs. -IAA

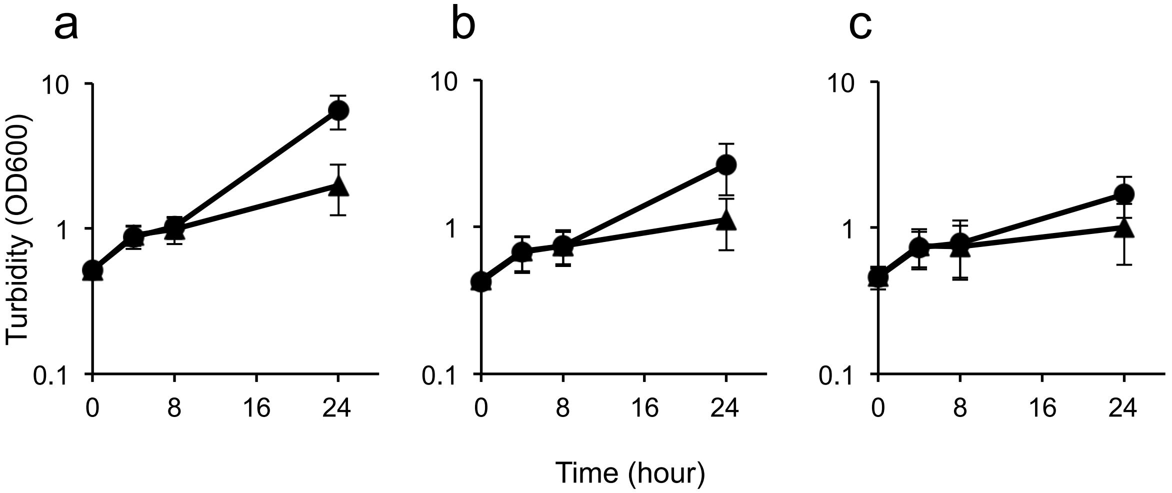

Supplementary Figure S2. Effect of overexpression of Kar9 and Bim1 on the growth of yeast cells.

Cells with empty vector (a), overexpressing Kar9 (b) or Bim1 (c) were incubated in SG broth with (triangle) or without (circle) 0.5% IAA. Empty vector-transformed cells were used as a control. Cell proliferation was assessed as turbidity of the cell suspension at 600 nm.

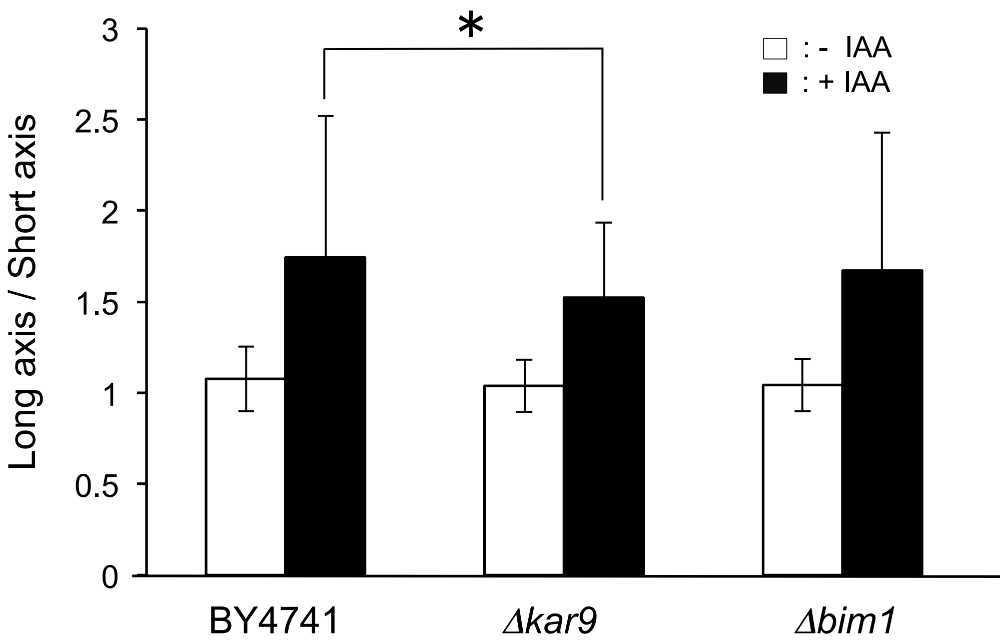

Supplementary Figure S3. Effect of IAA on morphology of Δ*kar9* and Δ*bim1*.

After 8-h incubation with or without 0.5% IAA, the lengths of the long and short axes of cells were measured. Data are means ± standard deviations of triplicate experiments. In each experiment, the cells were selected at random (n > 200). *$P < 0.05$ vs. Δ*kar9*.

Supplementary Table S1. *S. cerevisiae* strains used in this study

Yeast strains	Relevant genotype	Source
BY4741	*MATa his3Δ1 leu2Δ0 met15Δ0 ura3Δ0*	National BioResource Project - Yeast
W303-1A	*MATa leu2-3,112 trp1-1 can1-100 ura3-1 ade2-1 his3-11,15*	National BioResource Project - Yeast
BY23323	*MATa spc42::SPC42-tem1-3-HIS3 nud1-44 TUB1-GFP-URA3*	National BioResource Project - Yeast
BY24051	*MATa myo2::MYO2-GFP::TRP1 ura3-52 lys2-801 ade2-101 trp1-Δ63 his3-Δ200 leu2-Δ1*	National BioResource Project - Yeast
EY0986/GFP-Bim1	*MATa his3Δ1 leu2Δ0 met15Δ0 ura3Δ0 GFP-BIM1*	Life Technologies, Carbad, CA USA
EY0986/GFP-Bud6	*MATa his3Δ1 leu2Δ0 met15Δ0 ura3Δ0 GFP-BUD6*	Life Technologies, Carbad, CA USA
EY0986/GFP-Kar9	*MATa his3Δ1 leu2Δ0 met15Δ0 ura3Δ0 GFP-KAR9*	Life Technologies, Carbad, CA USA
BY4741	BY4741 harboring *pYES2*	This study
BY4741	BY4741 harboring *pYES2-KAR9*	This study
BY4741	BY4741 harboring *pYES2-BIM1*	This study
Δ*kar9*	*MATa his3Δ1 leu2Δ0 met15Δ0 ura3Δ0kar9::G418*	Open Biosystems
Δ*bim1*	*MATa his3Δ1 leu2Δ0 met15Δ0 ura3Δ0bim1::G418*	Open Biosystems
TUB2-DAmp	BY4741 haploid DAmP *TUB2*	GE Dharmacon, USA

Supplementary Table S2. Primer sequences for cloning of *KAR9* and *BIM1* into the vector pYES2 and primers used in RT-PCR.

**Name**	**Sequence**
KAR9-forward	ATTAAGCTTGGTACCGAGCTCAAAAAAATGGATAATGATGGACCCAGA
KAR9-reverse	TGATGCGGCCCTCTAGATTAATAAGTTGGGGTTTTATCTAAAC
BIM1-forward	ATTAAGCTTGGTACCGAGCTCAAAAAAATGAGTGCGGGTATCGGAG
BIM1-reverse	TGATGCGGCCCTCTAGATTAAAAAGTTTCCTCGTCGATG
Cytoskeleton	
TDH1-forward	5'- GGTGCCAAGAAGGTTGTCAT-3'
TDH1-revese	5`- CCTTAGCAGCACCGGTAGAG-3'
TUB1-forward	5'-ACTTACAACTGCAAACAAACA-3'
TUB1-reverse,	5'-AGAAAGGATAAGGAGGTTGGG-3'
TUB2-forward	5'-CAAAAGCAAAATCTCCACA-3'
TUB2-reverse	5'-GAAAAAGAAAGGAAAGCAAC-3'
TUB3-forward	5'-TAAGCAAGCGACTTGAGAC-3'
TUB3-reverse	5'-TATCGGGATCAGAGCCACTTT-3'
TUB4- forward	5'-AGGCCATTCCAGTTATGACG-3'
TUB4-reverse	5'-TTGCACGCATTCTCAAAGAC-3'
ACT1-forward	5'-ATGGTCGGTATGGGTCAAAA-3'
ACT1-reverse	5'-AACCAGCGTAAATTGGAACG-3'
Modification of mRNA	
CDC33-forward	5'-CTGAGCCACACGAACTACCA-3'
CDC33- reverse	5'-GGGTCTACCATTGGCACT-3'
PAB1-forward	5'-AGATGACAGCGTTGATGACG-3'
PAB1- reverse	5'-CCGTTGAATGGAACACCTCT-3'
EAP1-forward	5'-TAGCCAAAAAGGGCAGAGAA-3'
EAP1- reverse	5'-AACTGCCTCCACCATTGTTC-3'
CAF20-forward	5'-TGTTGGTCATTTCGGTCGTA-3'
CAF20– reverse	5'-ATGCTTCGTCGTCTTCGTCT-3'
Translation	
GCN3-forward	5'-ATGGCCAGCTTTTTGTATCG-3'
GCN3- reverse	5'-CTGATTCAGCAACACACCCTCA-3'
GCD6-forward	5'- TTGGAAGGAACTGCCAAATC-3'
GCD6- reverse	5'-ATCGTCAGACACCTCGTTT-3'
GCD1-forward	5'-TTGCCCATCGGTAATAGACC-3'
GCD1- reverse	5'-CGACCAAGACTTGTGGAGGT-3'
GCD7-forward	5'-GCCATTCAGGGTATCAAGGA-3'
GCD7- reverse	5'-AACGGCTTTAGTGCCATAA-3'
GCD2-forward	5'-CACTATTTGAAGGGCGGAAA-3'
GCD2- reverse	5'-ACCAGGTCATTTGGATCGC-3'
ANB1-forward	5'-CTTCCAAGACCGGTAAGCAC-3'
ANB1- reverse	5'-GAGATGGCAGCTTCTTCACC-3'
HYP2-forward	5'-AAGCACGGTCACGCTAAAGT-3'
HYP2- reverse	5'-CGGTTCTAGCAGCTTCCTTG-3'
Ubiquitin	
UBI4-forward	5'-CCTCCGACACGATCGATAAC-3'
UBI4-reverse	5'-TCTCAACACCAAATGCAACG-3'
CDS primer	5'-AAGCAGTGGTAACAACGCAGAGTAC TTTTTTTTTTTTTTTTTTTTTTTTTTTTTVN-3'
